# Trigeminal nerve stimulation: a current state-of-the-art review

**DOI:** 10.1186/s42234-023-00128-z

**Published:** 2023-12-13

**Authors:** Keren Powell, Kanheng Lin, Willians Tambo, Andrea Palomo Saavedra, Daniel Sciubba, Yousef Al Abed, Chunyan Li

**Affiliations:** 1 Translational Brain Research Laboratory, The Feinstein Institutes for Medical Research, 350 Community Dr, Manhasset, NY 11030 USA; 2 Institute for Bioelectronic Medicine, The Feinstein Institutes for Medical Research, Manhasset, NY USA; 3 Emory University, Atlanta, GA USA; 4 Elmezzi Graduate School of Molecular Medicine, The Feinstein Institutes for Medical Research, Manhasset, NY USA; 5 University of Notre Dame du Lac, Notre Dame, Notre Dame, IN USA; 6 Department of Neurosurgery, Zucker School of Medicine at Hofstra/Northwell, Hempstead, NY USA

**Keywords:** Trigeminal nerve stimulation, Trigeminal nerve, Vasoactive neuropeptide, Neurotransmission, Autonomic nervous system, Cerebral vasodilation, Cerebral blood flow, Bioelectronic medicine, Neuromodulation, Diving reflex

## Abstract

Nearly 5 decades ago, the effect of trigeminal nerve stimulation (TNS) on cerebral blood flow was observed for the first time. This implication directly led to further investigations and TNS’ success as a therapeutic intervention. Possessing unique connections with key brain and brainstem regions, TNS has been observed to modulate cerebral vasodilation, brain metabolism, cerebral autoregulation, cerebral and systemic inflammation, and the autonomic nervous system. The unique range of effects make it a prime therapeutic modality and have led to its clinical usage in chronic conditions such as migraine, prolonged disorders of consciousness, and depression. This review aims to present a comprehensive overview of TNS research and its broader therapeutic potentialities. For the purpose of this review, PubMed and Google Scholar were searched from inception to August 28, 2023 to identify a total of 89 relevant studies, both clinical and pre-clinical. TNS harnesses the release of vasoactive neuropeptides, modulation of neurotransmission, and direct action upon the autonomic nervous system to generate a suite of powerful multitarget therapeutic effects. While TNS has been applied clinically to chronic pathological conditions, these powerful effects have recently shown great potential in a number of acute/traumatic pathologies. However, there are still key mechanistic and methodologic knowledge gaps to be solved to make TNS a viable therapeutic option in wider clinical settings. These include bimodal or paradoxical effects and mechanisms, questions regarding its safety in acute/traumatic conditions, the development of more selective stimulation methods to avoid potential maladaptive effects, and its connection to the diving reflex, a trigeminally-mediated protective endogenous reflex. The address of these questions could overcome the current limitations and allow TNS to be applied therapeutically to an innumerable number of pathologies, such that it now stands at the precipice of becoming a ground-breaking therapeutic modality.

## Introduction

**Fig. 1:**
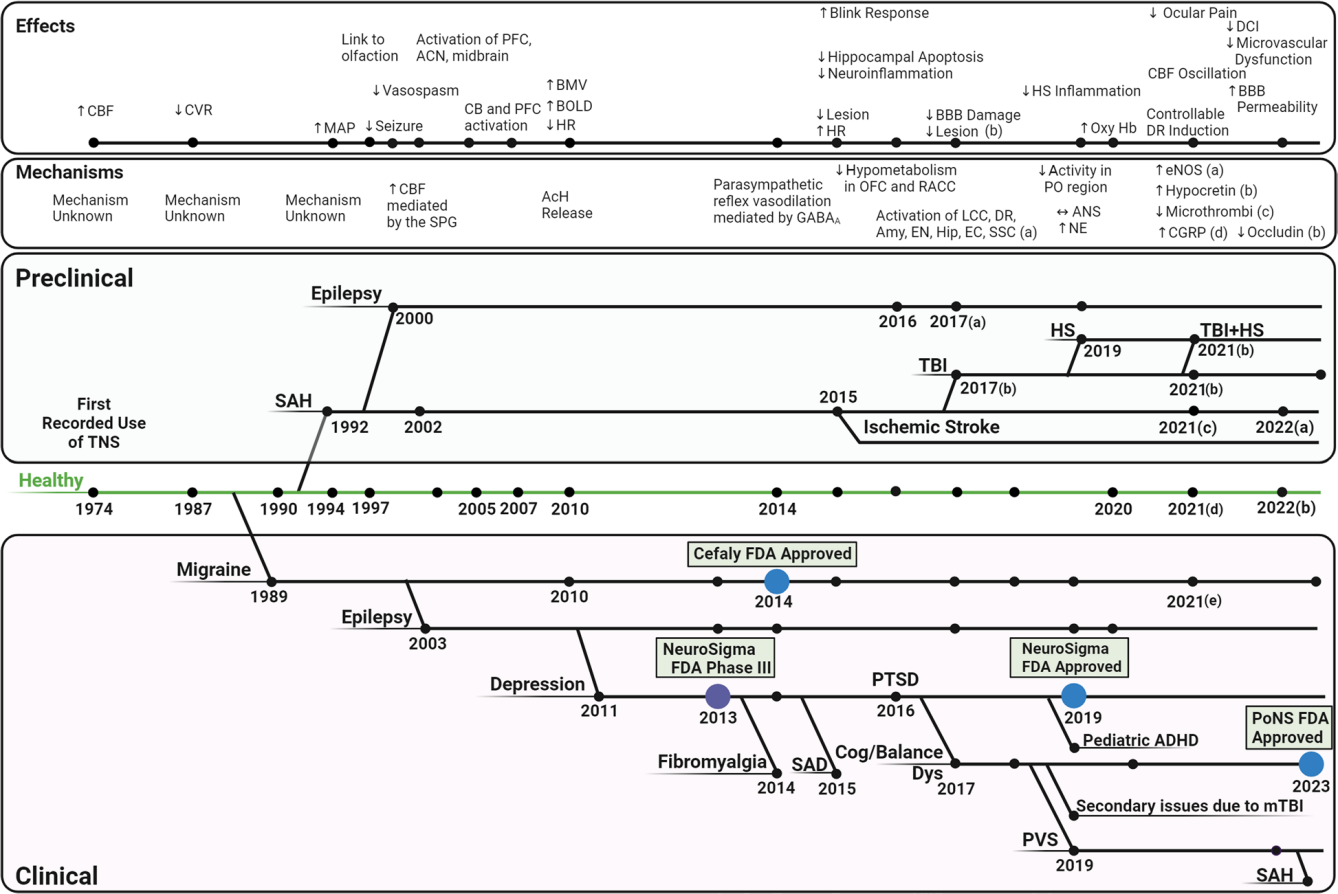
A brief history of Trigeminal Nerve Stimulation. TNS was first used in 1974 to assess the neural control of cerebral blood flow. Since then, it has been applied clinically in migraine, epilepsy, depression, PTSD, fibromyalgia, SAD, cognitive/balance dysfunction, pediatric ADHD, PVS, SAH, and secondary issues due to mTBI. In 2014, Cefaly was approved by the FDA for treatment of migraine, Neurosigma was approved in 2019 for pediatric ADHD, and PoNS was approved in 2023 for cognitive/balance dysfunction. Preclinically, the efficacy of TNS has additionally been assessed in TBI, HS, TBI + HS, and ischemic stroke. In the past decade, the effects and mechanisms of TNS within the brain and throughout the body have been expanded upon. (This figure was generated using BioRender.com) (AcH: acetylcholine: ACN: anticorrelated networks; ADHD: attention deficit and hyperactivity disorder; ANS: autonomic nervous system; BBB: blood–brain barrier; BOLD: Blood Oxygenation Level Dependent; CB: cingulum bundle; CBF: cerebral blood flow; CGRP: Calcitonin gene-related peptide; CVR: cerebrovascular resistance; HR: heart rate; HS: hemorrhagic shock; mTBI: mild traumatic brain injury; PFC: prefrontal cortex; PTSD: post-traumatic stress disorder; PVS: persistent vegetative state; SAD: social anxiety disorder; SAH: subarachnoid hemorrhage; TBI: traumatic brain injury; TNS: trigeminal nerve stimulation;)

Trigeminal nerve stimulation (TNS) dates back 5 decades, with an examination into the neural control of cerebral blood flow (CBF) (Lang and Zimmer 1974) (Fig. 1). Application of TNS resulted in a stronger increase in CBF than either vagal or sympathetic nerve stimulation, though the underlying mechanism was unknown. More than a decade later, cerebral vasodilation was identified as a possible factor (Goadsby and Duckworth 1987). These findings indicate that the application of TNS in cerebrovascular diseases has clinical potential in the field of bioelectronic medicine. Bioelectronic medicine is the practice of applying stimulus to the nervous system to harness electrical signaling, engendering localized and systemic interventions in pathological conditions (Singh et al. 2022; Denison and Morrell 2022; Pavlov and Tracey 2019; Sanjuan-Alberte et al. 2018; DeGiorgio et al. 2011; Gildenberg 2005). The therapeutic potential of TNS capitalizes on its unique connections to key brain regions, including the cerebrovasculature, limbic system, entorhinal cortex, trigeminal mesencephalic nucleus (TMN), and medullary dorsal horn (MDH) (Bathla and Hegde 2013; Degiorgio et al. 2011; Borges and Casselman 2010; Shankland 2000; Walker 1990). Neural impulses along these connections give rise to localized effects within the central nervous and cerebrovascular systems, as well as systemic effects, influencing symptomology in cerebral and systemic pathologic conditions.

TNS has been observed to induce cerebral vasodilation, modulate cerebral metabolism and neurotransmission, and decrease neuroinflammation (Xu et al. 2023; Nash, Powell, White, et al. 2023; Shah et al. 2020; Chiluwal et al. 2017; Magis et al. 2017; Weber et al. 2003). Systemically, it modulates the autonomic nervous system (ANS), induces peripheral vasoconstriction, decreases systemic inflammation, and modulates cardiac performance (Degiorgio et al. 2011; Borges and Casselman 2010; Shankland 2000; Walker 1990). Clinical and pre-clinical assessments in healthy conditions illustrate TNS’ widespread effects and the underlying mechanisms, forming the basis of its application in pathological conditions (Gong et al. 2022; Li et al. 2021a, b; Just et al. 2010; Weber et al. 2003; Suzuki et al. 1990). Clinically, TNS has been primarily applied to chronic brain disorders. It was first applied clinically to cluster headache in 1989 (Fanciullacci et al. 1989), followed by drug-resistant epilepsy in 2003 (Degiorgo et al. 2003). TNS is now being assessed as a treatment for drug resistant epilepsy (Gil-Lopez et al. 2020), depression (Cook et al. 2016 and 2013), post-traumatic stress disorder (PTSD) (Cook et al. 2016; Trevizol et al. 2015), prolonged disorders of consciousness (pDOC) (Ma et al. 2023), and long-term recovery following subarachnoid hemorrhage (SAH) (Rigoard et al. 2023). The success of TNS is illustrated in its FDA approval for the treatment of migraine (Vecchio et al. 2018; Chou et al. 2019; Reed et al. 2010), pediatric attention deficit and hyperactivity disorder (ADHD) (McGough et al. 2019 and 2015) and sensorimotor/cognitive dysfunction due to mild TBI (mTBI), multiple sclerosis (MS), and cerebral palsy (Ptito et al. 2021; Tyler et al. 2019; Ignatova et al. 2018) (Fig. 1). In pre-clinical studies, in addition to chronic brain disorders, TNS has demonstrated pronounced cerebro-protective properties, particularly in the hyper-acute phase, in conditions such as traumatic brain injury (TBI) (Chiluwal et al. 2017), hemorrhagic shock (HS) (Li et al. 2019), SAH (Li et al. 2021a, b; Shah et al. 2022; Salar et al. 1992), and ischemic stroke (Zheng et al. 2020; Shiflett et al. 2015). The current breadth of TNS research indicates that it is at a tipping point, with the capacity to emerge as a leading treatment, potentially poised to become as widespread as vagus nerve stimulation (VNS).

This systematic review aims to portray, for the first time, a holistic view of the state-of-the-art TNS research. Here, we first discuss the current clinical and pre-clinical understanding of the anatomical connections and recruitment of TNS. We then outline the role of TNS’ unique and powerful suite of effects and mechanisms which form the basis for the treatment of a multitude of chronic and acute/traumatic brain disorders. Next, we explore some of the key mechanistic and methodologic knowledge gaps to be addressed to aid the comprehensive translational potential of TNS, including bimodal and paradoxical effects and mechanisms, potential issues regarding its clinical translation to acute/traumatic conditions, adoption of more selective stimulation methods, and its relation to the diving reflex (DR), a trigeminally-mediated protective endogenous reflex. Lastly, we present a discussion of TNS’ wider therapeutic potential in central nervous system and autonomic disorders, as well as the diagnostic potential born out of the trigeminal nerve’s unique connections.

## Methods

### Data sources and searches

**Fig. 2:**
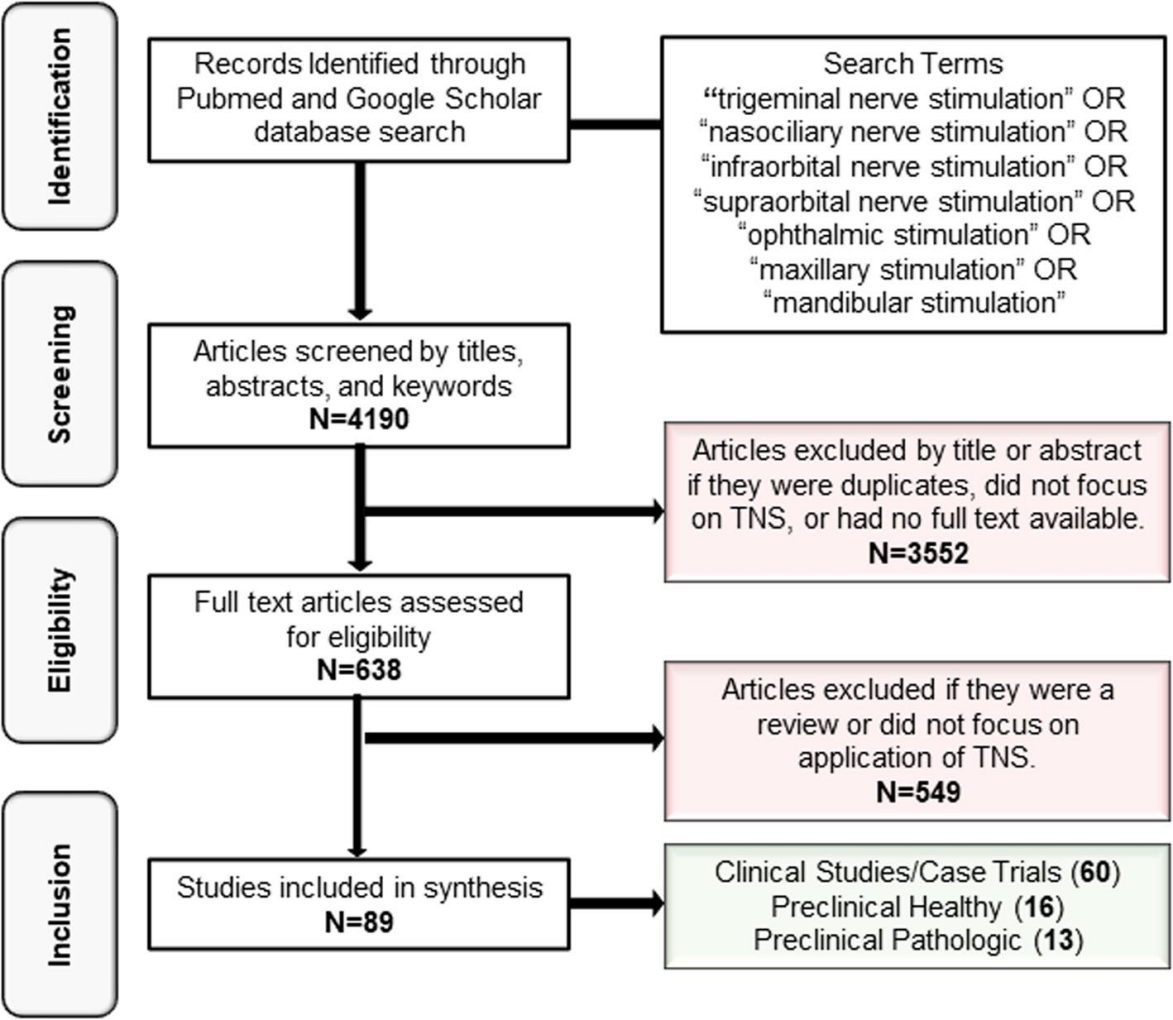
PRISMA flowchart of the identification, screening, and eligibility inclusion for papers included in this assessment

Data for this review were gathered from PubMed and Google Scholar from inception to August 16, 2023 (Fig. 2). The search terms were [“trigeminal nerve stimulation” OR “nasociliary nerve stimulation” OR “infraorbital nerve stimulation” OR “supraorbital nerve stimulation” OR “ophthalmic stimulation” OR “maxillary stimulation” OR “mandibular stimulation”].

### Eligibility criteria

We included all primary articles which discussed the application of TNS. The searches included studies, abstracts, and letters to the editor. Articles for which full-text copies could not be accessed, did not focus on TNS application, or were reviews were excluded. Using the designated search terms and criteria, 638 eligible articles were initially identified, and after abstract screening, 89 full-text articles were included. Of these articles, 60 were clinical studies or case trials (Table 1), 16 were pre-clinical studies performed in healthy animals (Table 2), and 13 were pre-clinical studies performed in pathological models (Table 3). Studies that were not indexed in either PubMed or Google Scholar were not included in this review.

**Table 1:** Clinical usage of TNS

Status	Medical Condition	Stimulation Method	Population	Stimulation Parameters	Observations	Adverse Events	Reference
**Clinical Usage**	Migraine	Infratrochlear nerve	Healthy, common migraine, episodic CH, and inactive phase of CH	40 mA 0.8 ms PW	Significant ipsilateral pupillary miosis	None stated	Fanciullacci et al. 1989
		Subcutaneous supraorbital and occipital nerve	Patients with CM	Not stated	↓ Pain and neurological symptoms	None stated	Reed et al. 2010
		Bilateral supratrochlear and supraorbital nerves with Cefaly	Migraine with or without aura	60 Hz 16 mA 0.25 ms PW 20 min/day	↓ Migraine severity and frequency	None stated	Schoenen et al. 2013
		Cefaly—Transcutaneous Supraorbital Nerve	Chronic Migraine, 60% with medication overuse	100 Hz 16 mA 0.25 ms Pw	↓ Muscle EMG	None stated	Didier et al. 2015
				60 Hz 16 mA 0.3 ms PW			
		Cefaly—Transcutaneous Supraorbital Nerve	67 episodic migraine patients	60 Hz 16 mA 0.25 ms PW 20 min per day	↓ Migraine days	Paresthesia reported	Reiderer et al. 2015
		Cefaly supraorbital and supratrochlear stimulation	24 patients with migraine without aura	60 Hz 16 mA 0.25 ms PW 20 min/day	↓ Migraine severity and frequency ↓ HIT-6 questionnaire rating	None stated	Russo et al. 2015
		3 months Cefaly—Transcutaneous Supraorbital Nerve	28 individuals Episodic Migraine	60 Hz 16 mA 0.25 ms PW 20 min daily for 3 months	↓ Migraine days ↓ Hypometabolism in OFC and rACC	None stated	Magis et al. 2017
		Cefaly eTNS	30 patients diagnosed with migraine with or without aura	100 Hz 16 mA 0.25 ms PW 60 min/day	↓ Pain intensity	None stated	Chou et al. 2017
		Cefaly	Chronic migraine	60 Hz 16 mA 0.25 ms PW 20 min/day	↓ Frequency ↓ Medication usage	None stated	Di Fiore et al. 2017
		Cefaly—Transcutaneous Supraorbital Nerve	109 Participants	100 Hz 16 mA 0.25 ms PW 1 h	↓ Pain scores	None stated	Chou et al. 2019
		Transcutaneous supraorbital nerve stimulation (Cefaly)	Migraine w/out aura	60 Hz 16 mA 0.25 ms PW 20 min/day	Strong placebo effect was noted	None stated	Vecchio et al. 2018
		Cefaly on supratrochlear and supraorbital of ophthalmic	Vestibular migraine	60 Hz 16 mA 0.25 ms PW 20 min/day	↓ Vertigo and headache ↓ Symptoms	None stated	Beh 2020
		Supraorbital and supratrochlear nerve electrostimulation	25 participants with CM	60 Hz 16 mA 0.25 ms PW 20 min/day	↑ Sleep ↓ Pain severity and frequency	Some paresthesia, unpleasant dysesthesias, mild dizziness, mild nausea, somnolence	Ordas et al. 2020
		Cefaly eTNS	Patients with CM	100 Hz 16 mA 0.25 ms PW 120 min/day	↓ Headaches	Only minor AEs, such as paresthesia	Kuruvilla et al. 2019
		Cefaly eTNS	18 patients with ocular pain with/without CM	60 Hz 16 mA 0.25 ms PW 20 min/day	↓ Ocular pain intensity ↓ Light sensitivity ↓ Wind sensitivity ↓ Burning sensation	Sedation	Mehra et al., 2021
		Cefaly eTNS	24 patients with CM	60 Hz 16 mA 0.25 ms PW 20 min/day	↓ Headache days	None stated	Trimboli et al. 2023
	pediatric ADHD	8-week NeuroSigma Monarch eTNS – bipolar V1 stimulation	Adolescents aged 7–14 with DSM IV ADHD	120 Hz 0.25 ms PW 30 s on 30 s off 7 – 9 h per night	↓ Inattentive and hyperactivity ↑ Height, weight, pulse	None stated	McGough et al. 2019
	Balance difficulties due to mTBI	Helius Medical PONS-Translingual eTNS	Adults	Not stated	↓ SOT, DGI, and 6MWT scores	2 mild and 6 moderate AEs	Tyler et al 2019
		Translingual neurostimulation	Adults with mTBI diagnosis and balance deficit	High Freq: 150 Hz 0.4—60 µs PW 5 ms interval	↑ DGI scores ↓ HDI and SQI	None stated	Ptito et al. 2021
				Low Freq: 0.08 Hz 12.5 s interval			
**Clinical Trials/ Testing**	Drug Resistant Epilepsy	Unspecified	2 patients	120 Hz 8 – 25 mA 20 – 30 s on 20 – 30 s off 24 h	↓ Seizures	None stated	DeGiorgio et al. 2003
		NeuroSigma Monarch eTNS – bipolar stimulation at ophthalmic and supratrochlear nerves	Patients with 2 or more partial onset seizures per month	120 Hz 0.25 ms PW	↓ In seizure frequency Mood improvement	Anxiety, headache, and skin irritation	DeGiorgio et al. 2013
			Patients with DRE	120 Hz 7/30 s > 8 h/day	↑ Quality of life	No severe adverse events	Slaght et al. 2017
			Patients with DRE, use of at least 1 AED	Not stated	↑ Sleep, mood, and energy ↓ Seizures	None stated	Olivie et al. 2019
			Patients with DRE	120 Hz < 10 mA 0.25 ms PW 30 s on 30 s off At least 8 h/day	↑ Quality of life ↓ Seizure rates	No severe adverse events	Gil-Lopez et al. 2020
		Unspecified	Patient with DRE which progressed to RSE	None stated	Improved mental status ↓ Seizures	No severe AE	Moseley et al. 2014
		APEX set for neuromodulation	Patients with DRE	120 Hz 0.25 ms PW 5 s on 5 s off	↓ Seizures	No severe AE	Zare et al. 2014
		Winner stimulator	29 subjects with DRE	120 Hz 1 – 20 mA 0.25 ms PW 30 s on 30 s off 20 min	↑ Absolute power of alpha band in the parietal-occipital areas	None stated	Ginatempo et al. 2019
	Depression	8 weeks external trigeminal nerve stimulation (NeuroSigma predecessor)	Pharmacological resistant major depression	Not stated	↓ HDRS and BDI scores	None stated	Schrader et al 2011
		NeuroSigma Monarch eTNS	Adults with nonpsychotic unipolar MDD	120 Hz 4 – 6 mA 0.25 ms PW 30 s on 30 s off	↑ Quality of life ↓ Depression test scores	No severe adverse events	Cook et al 2013
			Patients diagnosed with MDD	120 Hz 0 – 100 mA 0.25 ms PW Cycle of 30 s	↓ Depression test scores	No severe adverse events	Shiozawa et al 2014a, b
		10 Days Ibramed Neurodyn eTNS- V1 TENS stimulation	Patients between 18 and 69 with severe MDD	120 Hz 0 – 100 mA 0.25 ms PW Cycle of 30 s	↓ Depressive symptoms	None stated	Generoso et al 2019
	Fibromyalgia	10 days of bilateral supraorbital stimulation	Patient with fibromyalgia	120 Hz 0.25 ms PW 30 min	↓ Pain ↓ Depressive symptoms	None stated	Shiozawa et al. 2014a, b
	Social Anxiety Disorder (SAD)	10 days supraorbital electrostimulation	Patient with SAD	120 Hz 0.25 ms PW 30 min continuous	↓ Avoidance behaviors ↓ SPIN and LSAS scores	None stated	Trevizol et al. 2015
	PTSD	Bilateral trigeminal nerve electrostimulation	5 patients with PTSD and MDD	120 Hz 0.25 ms PW 30 min/day	↓ PTSD and depressive symptoms	No serious AE reported	Trevizol et al 2015
		NeuroSigma Monarch eTNS	Adults with both PTSD and MDD, 18–75 in age	120 Hz 4 – 6 mA 0.25 ms PW 30 s on 30 s off 8 h each night	↓ PCL score	None stated	Cook et al 2016
	Cognitive/ Sensory Dysfunction Secondary to Other Pathologies	Portable Neuromodulation Stimulator (PoNS™)	14 MS patients	Not stated	↑ SOT scores ↑ BOLD in bilateral premotor areas	None stated	Leonard et al. 2017
		EA/TNS for 15 acupoints (some on trigeminal facial nerves)	Females 18–65 with breast cancer, either undergoing or finished chemo less than 2 weeks ago	2 Hz 6 V, 48 mA 0.1 ms PW 30 min	↓ Diarrhea, tenseness, worriness, irritation, headache, and tinnitus	None stated	Zhang et al. 2020
	Persistent Vegetative State	Ophthalmic nerve and maxillary nerve	Patients in PVS	40 Hz 18 – 20 mA 0.2 ms PW 30 s/min 6 h/day	Spontaneous eye opening and exclamatory articulated speech after 4 weeks of TNS ↑ GCS scores	None stated	Fan et al. 2019
		Suborbital foramen and bilateral superior orbital fissures	Patients with Disorders of Consciousness	40 Hz 18 – 20 mA 200 ms PW 30 s/min	Signs of recovery	None stated	Dong et al. 2022
		Bilateral maxillary and mandibular nerve	Patients with DOC after suffering TBI	40/28 Hz 8 mA 40 min	↑ CRS-R scores	None stated	Wu et al. 2022
		Bilateral supraorbital foramen and infraorbital foramen	Patients in PVS	40 Hz 10 – 15 mA 0.2 ms PW 30 s/min 3 h/day	↑ Recovery ↑ Total GCS and CRS-S scores ↓ Hypometabolic areas	None stated	Ma et al. 2023
	Subarachnoid Hemorrhage	Supratrochlear and supraorbital transcutaneous electrical stimulation	Patients with SAH	20 Hz 14 days Continuous	No significant effects on vasospasm-related DCI, functional outcome, and health-related quality of life	None stated	Rigoard et al. 2023
	Parkinson’s Disease	Gaseous CO_2_	Healthy adults and Patients with olfactory dysfunction due to PD	Unilateral nasal exposure 200 ms 145 mL/s	Trigeminal olfactory event related potentials were the same between healthy and PD individuals	None stated	Barz et al. 1997
		Eucalyptol	Healthy adults and Patients with olfactory dysfunction due to PD	Unilateral nasal exposure	Trigeminal sensitivity was the same in healthy and PD individuals	None stated	Tremblay et al., 2017
	Healthy	nuroStym device at the right forehead and cheek	Healthy adults	350 Hz 3.5 mA 10% DC 20 min	Slight decrease in creatine concentrations in the DLPFC region	None stated	Ritland et al. 2023
		Minimally Invasive Electroacupuncture, Supraorbital nerve	Healthy adults	100 Hz 0.1—0.2 mA 0.25 ms PW 5 cycles of 1 min on 1 min off for 15 min	↑ CBF in the prefrontal cortex ↑ Oxygenated Hb in PFC ↓ Deoxy Hb in R-PFC ↓ HR w/ EA	None stated	Suzuki et al. 2020
		Ophthalmic nerve	Healthy adults	120 Hz 30 s/min 30 min continuous	↓ Mean olfactory detection threshold ↓ Sleepiness	None stated	Badran et al. 2022
		Ophthalmic nerve	Healthy adults	3 kHz 50 ms PW 30 s/min 20 min	No significant effect on learning	None stated	Arias et al. 2022
		Opthalmic nerve + Transcranial Magnetic Stimulation	Healthy adults	120 Hz 1.8–6.8 mA 0.25 ms PW 40 min	↔ Cortical excitability	None stated	Axelson et al. 2014
		Infraorbital nerve	17 Healthy adults	120 Hz 1 – 20 mA 0.25 ms PW 30 s on 30 s off for 20 min	↓ R2 area ↔ R1 area ↔ R2 recovery cycle ↔ Intracortical excitability	None stated	Mercante et al. 2015
		Infraorbital nerve	20 Healthy adults	120 Hz 1 – 20 mA 0.25 ms PW 30 s on 30 s off for 20 min	R2 blink response inhibited up to 60-min after TNS	None stated	Pilurzi et al. 2016
		Infraorbital nerve	18 Healthy adults	120 Hz 1 – 20 mA 0.25 ms PW 30 s on 30 s off for 20 min	↓ Beta-frequency intra- and interhemispheric coherences ↑ EEG frequency total power ↓ Interhemispheric gamma coherence	None stated	Ginatempo et al. 2018
		Infraorbital nerve	15 Healthy adults	120 Hz 6 – 18 mA 0.25 ms PW 30 s on 30 s off for 20 min	↓ Hand-blink reflex magnitude	None stated	Mercante et al. 2020
		Supraorbital nerve	Healthy adults	400 Hz Variable intensity	Blink potentiation for > 1 h	None stated	Mao and Evinger, 2001
		Infraorbital nerve	Healthy adults	120 Hz 1 – 20 mA 0.25 ms PW 30 s on 30 s off for 20 min	No significant increases in measured ERP components	None stated	Mercante et al. 2023
		Trigeminal stimulation with gaseous CO_2_	12 normosmic and 11 anosmic	Not stated	Activation of cerebellum and premotor cortex, PFC, anterior cingulum, cingulate gyrus, insula	None stated	Iannilli et al 2007
		CO_2_ trigeminal stimulation	19 healthy	N/A	Activation of insula, middle frontal gyrus, and supplemental motor area, midbrain, DOFC, frontal operculum, superior temporal gyrus, medial frontal gyrus, and anterior caudate nucleus	None stated	Hummel et al 2005
		Facial cooling	Healthy adults	N/A	↓ HR	None stated	Janczak et al. 2022
		Facial cooling	Healthy adults	N/A	↑ CBV ↓ HR ↑ BP ↑ Coronary circulation	None stated	Prodel et al 2023

**Table 2:** Preclinical usage of TNS in healthy conditions

Stimulation Method and Target	Species	Stimulation Parameters		Effect	Reference
Electrical, Distal Trigeminal Ganglion	15 isolated canine brains	50 Hz 10 V 1 ms PW 20 ms train		↑ CBF; ↓CVR ↑ Cerebral oxygen consumption	Lang and Zimmer 1974
Electrical, Trigeminal Ganglion	Adult cats	10 Hz 0.5 mA 0.25 ms PW		↑ CBF in the frontal and parietal cortex Neurally controlled cortical vasodilation	Goadsby and Duckworth 1987
	Male Wistar rats	5 Hz 10 V 1 ms PW 30 s continuous		Small increase in MAP and HR	Escott et al. 1995
	Cats	0.5 – 20 Hz 0.25 mA 0.25 ms PW 30 s continuous		↓ CVR in the parietal cortex ↓ BP	Goadsby et al. 1997
	Male Sprague–Dawley rats	1 mA 200 ms cycle 5 ms width 30 min continuous		↑ Plasma and cortical CGRP, PACAP, VIP, NPY, nociceptin	Guo et al. 2021
Invasive Electrical, Right Nasociliary Nerve	Adult Sprague–Dawley rats	3 – 60 Hz 5 V 0.5 ms PW 90 s continuous		↑ CBF as a function of frequency in the parietal cortex	Suzuki et al. 1990
Non- Invasive Electrical, Nasociliary Nerve	New Zealand albino rabbits of both sexes	10 Hz 5 V 0.5 ms PW 90 s continuous		↑ CBF as a function of voltage (1-5 V) in the premotor cortex	Gurelik et al. 2004
Percutaneous Electrical, Infraorbital Nerve	Male Sprague–Dawley rats	2 Hz 1.2 mA 1 ms PW 4 min continuous		↑ CBF in the barrel cortex Pronounced increase in K1 and K2 of the H_2_^15^O, which correlates to ↑ CBF	Weber et al. 2003
	15 male Sprague–Dawley rats	0.25—12 Hz 2 mA 0.1 ms PW		↑ CBF as a function of frequency (up to 3 Hz) in the barrel cortex Activation of somatosensory barrel field cortex above .5 Hz Blood oxygen response reached maximum at 1 Hz and/or 1.3 mA ↓ BP above 3 mA	Just et al. 2010
	Male Sprague–Dawley rats	50 Hz 0.25 – 5 V .75 ms PW Rectangular biphasic pulses		↓ HR; ↑ MAP; ↓SPO_2_; ↑ rCBF; ↓ CVR; ↑ P_br_O_2_ ↓RR; Brainstem Fos activation	Shah et al. 2020
	Male Sprague–Dawley rats	133 Hz 0.25 – 3 V 1 ms PW Rectangular biphasic pulses 60 s continuous		↑ CBF; ↓ CVR; ↑ MAP; ↑ CGRP; ↑ CPP	Li et al. 2021a, b
Infraorbital nerve, electrical acupuncture	12-week old Sprague–Dawley Rats	2/100 Hz 3 mA 6 s continuous for 40 min		↑ BBB permeability ↓ Occludin ↑ Calcium wave events with ↑ intensity	Gong et al. 2022
Peripheral trigeminal nerve stimulation	Adult female Wistar rats	5 Hz 0.05 mA 1 ms PW 10 s continuous	1 Hz 1—6 mA 0.1 ms PW	Both Settings: ↑ CBF ↑ Blood mean velocity	Li et al. 2009
Maxillary and mandibular branches	Adult female and male rats	0.05—1.5 mA 0.2 ms PW Pseudorandom intervals of 3-4 s apart with different combinations		Both the Ion and SoN can be accessed through a single incision, and both can be accessed, interfaced, and engaged in rats	Dingle et al. 2019
Invasive Electrical, cut end of Lingual Nerve	Adult cats	10 Hz 30 V 2 ms PW 20 s continuous		↔ CBF; ↑ CCA BF	Sato et al. 1997
	Adult male Wistar rats	1 – 30 Hz 1 – 30 V 2 ms PW 20 s continuous		↑ ICABF and LBF	Ishii et al. 2014

**Table 3:** Preclinical usage of TNS in pathological conditions

Model	Stimulation Method and Target	Species	Stimulation Parameters	Effect	Reference
Epilepsy	Infraorbital unilateral or bilateral electrostimulation	Adult female Long-Evans hooded rats	C1 – 333 Hz 3 – 11 mA 0.5 ms PW	↓Seizure frequency and severity Bilateral stimulation is more effective than unilateral at the same intensity	Fanselow et al. 2000
	Bilateral percutaneous electrical on ophthalmic nerves	Adult male Sprague–Dawley rats	140 Hz 10 mA 0.5 ms PW 1 min on, 4 min off	↓ Hippocampal apoptosis ↓ Neuroinflammation	Wang et al. 2016
	Unilateral electrical left infraorbital nerve	Adult male Sprague–Dawley rats	30 Hz 2 mA 0.5 ms PW 30 s on, 5 min off	↑ Mild seizure duration ↓ Severe seizure duration Activation in Sol, LCC, DR, amygdala, endopiriform nucleus, hippocampus, entorhinal cortex, somatosensory cortex	Marcante et al., Mercante et al. 2017
Subarachnoid Hemorrhage with CBF reduction	Invasive Electrical	Pittmann-Moore pigs	45 Hz 0.2 ms PW 3 h continuous	↑ CBF; ↓ CVR	Salar et al. 1992
Subarachnoid Hemorrhage	Non- Invasive, Transcorneal, Nasociliary Nerve	Male albino Wistar rats	30 Hz 3 mA 1 ms PW 30 s continuous	↑ CBF at the MCA; ↓ CVR; ↑ ABP	Atalay et al. 2002
Subarachnoid Hemorrhage – Endovascular Puncture	Percutaneous Electrical, Infraorbital Nerve	Male Sprague–Dawley rats	50 Hz 0.5 – 2.5 V 20 s every 5 min for 60 min	↑ CBF; ↑ CSF CGRP ↓ Macro-and micro-vascular vasospasm ↓ Parenchymal microthrombi ↓ Apoptosis ↓ Neurobehavioral deficits	Shah et al., 2022
Ischemic Stroke—Middle Cerebral Artery Occlusion	Subcutaneous Electrical/Cold Temp Forehead Stimulation	Male Sprague–Dawley rats	25 Hz 0.06 mA 0.5 ms PW	Both: ↓ Focal ischemic infarctions Cold Temp: ↑ MAP; ↑ HR	Shiflett et al. 2015
Traumatic Brain Injury	Invasive and Subcutaneous Electrical	Male Sprague–Dawley rats	1 min on per 11 min	TBI: ↑ CBF; ↑ MAP; ↑ PP; ↑ HR; ↑ PbrO2; ↓ RR w/out TBI: ↑ CBF, ↓CVR	Chiluwal et al. 2017
	Electrical acupuncture on infraorbital nerve	B6 mice	10 Hz 0.1 mA 200 ms PW 30 s on 30 s off for 60 min	↓ Edema; ↑ BOLD ↓ Sensorimotor dysfunction ↑ WGMT scores ↓ Inflammation	Yang et al. 2022
	Infraorbital nerve	Mice	40 Hz 0.2 mA 200 ms PW 30 s on 30 s off for 60 min	↓ APP and Iba1 in HPC ↑ Dopaminergic activation	Xu et al. 2023
Traumatic Brain Injury-induced Loss of Consciousness	Bilateral electrical anterior ethmoidal nerve	Male Sprague–Dawley rats	140 Hz 1 mA 10 s on, 5 s off for 30 min	↑ Consciousness levels ↑ EEG voltage ↑ Hypocretin expression ↑ LH neuron activation Sp5 glutamatergic neuron activation	Zheng et al. 2021
Traumatic Brain Injury Complicated by Hemorrhagic Shock	Percutaneous electrical, infraorbital and anterior ethmoid branches of trigeminal nerve	Male Sprague–Dawley rats	50 Hz 5 s PW 20 ms between pulses 5 s on, 5 s off for 1 min every 10 min, for total of 60 min	CBF oscillation ↑ Survival rate ↓ Lesion volume ↑ eNOS; ↓ iNOS ↓ Neuroinflammation	Li et al. 2021a, b
Hemorrhagic Shock	Percutaneous Electrical, Infraorbital Nerve	Male Sprague–Dawley rats	25 Hz 7 V 0.5 ms PW	↑CBF ↑MAP ↑PbO2 ↑Survival time ↑Norepinephrine ↓Systemic inflammatory markers	Li et al. 2019

## Anatomical connections and recruitment

**Fig. 3:**
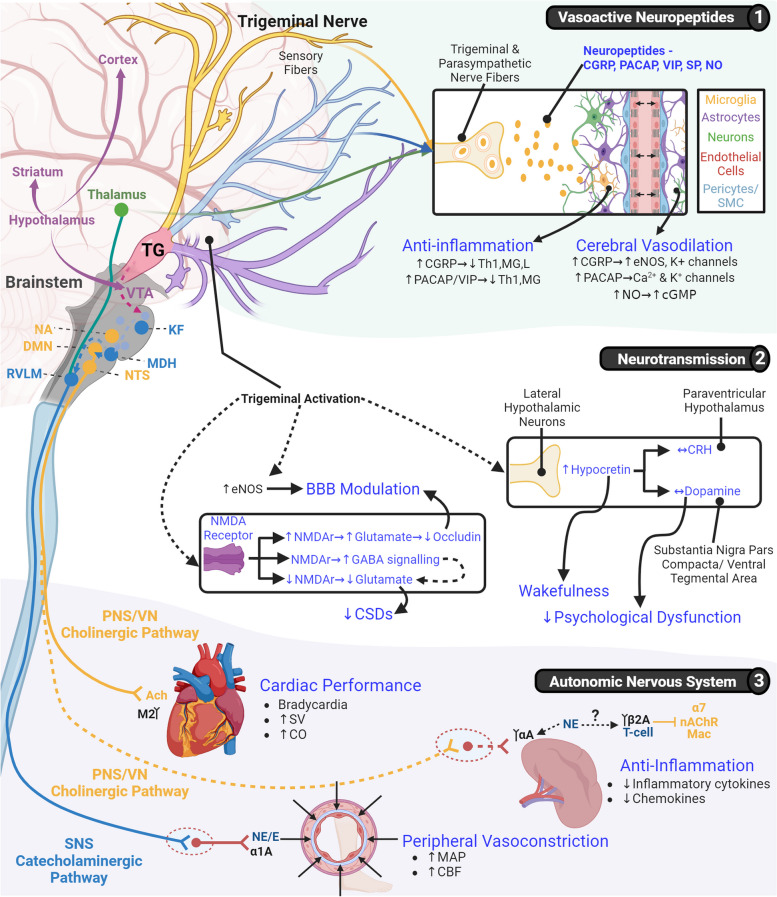
The underlying mechanisms of trigeminal nerve stimulation’s effects. The main mechanisms of TNS can be split into three main categories, 1)the release of vasoactive neuropeptides, 2)the modulation of neurotransmission, and 3)modulation of the ANS. The direct release of vasoactive neuropeptides such as CGRP, PACAP, VIP, SP, and NO from trigeminal and parasympathetic nerve fibers underlies the observed anti-inflammatory and cerebral vasodilatory effects of TNS. Via the increase of hypocretin and dopamine, there is an increase wakefulness and psychological dysfunction. The upregulation of eNOS and upregulation of NMDA receptors and glutamate result in bimodal modulation of BBB permeability, while the downregulation of NMDA receptors decreases glutamatergic transmission and increases GABA, thus modulating CSDs. Systemically, the cholinergic and catecholaminergic pathways of the ANS are harnessed by TNS. Through the cholinergic pathway of the PNS and upregulation of acetylcholine, cardiac performance is modulated when the acetylcholine binds to the muscarinic receptors on the heart. This cholinergic pathway is hypothesized to be the mechanism which gives rise to TNS’ systemic anti-inflammation as well. The released acetylcholine would stimulate α7-nicotinic receptors on splenic macrophages and decrease proinflammatory cytokine production. Concurrent upregulation of the SNS and the catecholaminergic pathway induces the release of norepinephrine peripheral vasoconstriction, leading to increased blood pressure and CBF. (This figure was generated using BioRender.com) (Ach: acetylcholine; ANS: autonomic nervous system; BBB: blood–brain barrier; CGRP: Calcitonin gene-related peptide; CO: cardiac output; CRH: corticotropin releasing hormone; DMN: dorsal motor nucleus; eNOS: endothelial nitric oxide synthase; GABA: gamma-aminobutyric acid; KF: Kölliker-FuseNO: nitric oxide; L: leukocyte; MAP: mean arterial pressure; MDH: medullary dorsal horn; MG: microglia; NA: nucleus accumbens; NE/E: norepinephrine/epinephrine; NMDA: N-methyl-d-aspartate; NTS: nucleus tractus solitarius; PACAP: pituitary adenylate cyclase-activating peptide; PNS: parasympathetic nervous system; RVLM: rostral ventrolateral medulla; SNS: sympathetic nervous system; SP: Substance P; SV: stroke volume; TG: trigeminal ganglion; TNS: trigeminal nerve stimulation; VIP: vasoactive intestinal peptide; VN: vagus nerve; VTA: ventral tegmental area)

The trigeminal nerve originates within the pons and divides at the trigeminal ganglion, forming the ophthalmic (V1), maxillary (V2), and mandibular (V3) branches (Fig. 3) (Bathla and Hegde 2013; Borges and Casselman 2010; Walker 1990). V1 gives rise to the frontal, lacrimal, nasociliary, tentorial, and dural nerves and V2 divides into the infraorbital, zygomatic, greater and lesser palatines, posterior superior alveolar, and meningeal nerves (Bathla and Hegde 2013). These branches are all sensory, providing touch, pain, and temperature input from the face to the central nervous system (CNS) (Walker 1990). V3, however, contains both sensory and motor nerves (Bathla and Hegde 2013). The meningeal, lingual, auriculotemporal, inferior alveolar, and buccal nerves of V3 are sensory; the masseteric, deep temporal, medial and lateral pterygoids, and mylohyoid nerves are motor (Bathla and Hegde 2013) and provide innervation to the muscles of the first branchial arch (Go et al. 2001).

Inputs from the trigeminal sensory afferents interact with the TMN and MDH brainstem nuclei (McCulloch et al. 2016; Panneton et al. 2000). This activates efferent signaling through the rostral ventrolateral medulla (RVLM), catecholaminergic regions (C1), locus coeruleus (LC), raphe nuclei (RN), nucleus tractus solitarius (NTS), Kölliker-Fuse (KF), and dorsal motor nucleus (DMN). In healthy conditions, TNS induces both long-term depression and long-term potentiation of brainstem interneurons within the arc of the corneal reflex, including the RN, LC, and NTS (Pilurzi et al. 2016; Mercante et al. 2015. Efferent trigeminal signaling feeds peripheral and central parasympathetic activity through the NTS and DMN, recruiting the vagus nerve which modulates cardiac performance and systemic inflammation (Panneton and Gan 2014; Burke et al. 2011; Panneton et al. 2006; Taylor et al. 2001; McCulloch et al. 1999). It mediates sympathetic responses through activation of the RVLM, C1, LC, and KF, resulting in peripheral vasoconstriction that upregulates systemic blood pressure (BP) and central blood flow.

TNS activates forebrain structures, including the entorhinal cortex, endopiriform nucleus, amygdala, and hippocampus (Mercante et al. 2018 and 2017). These structures are involved in the pathophysiology of CNS disorders for which TNS is currently under clinical assessment, including depression and epilepsy (Mercante et al. 2018; Cook et al. 2013; DeGiorgio et al. 2013; Schoenen et al. 2013; Fanselow et al. 2012). In patients with epilepsy, but not healthy individuals, TNS indirectly inhibits cortical activity and excitation in the parietal-occipital regions (Mercante et al. 2020; Ginatempo et al. 2019; Axelson et al. 2014). The electroencephalographic desynchronization induced by TNS in healthy conditions indicates that this may possibly mediated by actions upon the brainstem reticular formation (Ginatempo et al. 2018). Prolonged exposure to TNS has additionally been shown to mitigate migraine-induced hypometabolism in the orbitofrontal and rostral anterior cingulate cortices, which correlate to pain control (Magis et al. 2017). TNS in patients with pDOC not only reduces hypometabolism throughout the brain, but also produces a hypermetabolic response in the parahippocampal cortex, middle cingulate cortex, and precuneus regions (Ma et al. 2023). These areas relate to memory, recollection, autonomic functions, environmental perception, and cue reactivity, which could explain the effects in pDOC patients (Ma et al. 2023; Borsook et al. 2015; Aminoff et al. 2013; Treede and Apkarian 2008).

## Effects of trigeminal nerve stimulation

### Cerebral blood flow

TNS increases CBF in both healthy and pathological conditions (White and Powell et al. 2021; Suzuki et al. 1990). This increase has been observed, in pre-clinical models, to range from 15% (Suzuki et al. 1990) to upwards of 50% (Shah et al. 2020). TNS can increase CBF in different manners, including a rapid, direct increase (Weber et al. 2003; Suzuki et al. 1990), a gradual, graded increase (Shah et al. 2020), and in an oscillatory pattern (Li et al. 2021a, b(a)), to induce different effects suitable for specific disorders. The observation that the increase in CBF was independent of BP (Li et al. 2021a, b(b); Gürelik et al. 2004) and was not abrogated by parasympathetic blockade (Lang and Zimmer 1974), indicated cerebral vasodilation is a key underlying factor. This is supported by the observation that noninvasive TNS of the rat nasociliary nerve results in a 24–39% decrease in cerebrovascular resistance (CVR) (Atalay et al. 2002). However, the vasodilatory effect of TNS is not limited to only the macrovasculature; it also affects the microvasculature (Shah et al. 2022). Percutaneous TNS in an endovascular puncture model of SAH protects against pial arteriole constriction, with an ~ 30% decrease in vessel thickness, in addition to abrogating large vessel constriction in the internal carotid, middle cerebral, and anterior cerebral arteries.

### Anti-inflammatory effects

TNS decreases neuroinflammation in experimental models of epilepsy and trauma (Xu et al. 2023; Yang et al. 2022; Chiluwal et al. 2017; Wang et al. 2016). In epileptic rats, TNS reduces microglial activation and hippocampal concentrations of IL-1ß and TNF-α (Wang et al. 2016). Work by our lab has demonstrated that TNS in TBI reduces brain cortical TNF-α and IL-6 in rats (Chiluwal et al. 2017), while Yang et al. (2022) observed a decrease in cleaved caspase 3 expression in TBI. TNS also decreases hippocampal microglial activation in TBI, as indicated by decrease of Iba1 (Xu et al. 2023). The anti-inflammatory effect is further confirmed by the observation that TNS reduces microthrombi density by ~ 53% following SAH in rats (Shah et al. 2022). In SAH, microthrombi formation occurs when activated macrophages release pro-inflammatory chemokines and cytokines, which then induce adhesion molecule expression by endothelial cells (McBride et al. 2017). These activate circulating platelets and leukocytes, the former which aggregate and develop into thrombi which can occlude distal microvessels. Activated platelets and leukocytes feed back into each other, forming a thromboinflammatory feedback loop. Work by our lab additionally showed that TNS reduced serum levels of TNF-α and IL-6 after pre-clinical HS (Li et al. 2019), indicating a systemic anti-inflammatory effect as well.

### Blood–brain barrier

TNS has a bimodal effect on blood–brain barrier (BBB) permeability. In healthy animals, electroacupuncture TNS (EA-TNS) applied at the infraorbital nerve resulted in increased BBB permeability, as indicated by increased Evans-Blue perfusion (Gong et al. 2022). In a rat model of TBI, however, the application of TNS at the anterior ethmoidal nerve resulted in decreased BBB permeability (Chiluwal et al. 2017). The decrease of BBB permeability by TNS has been correlated to a decrease in VEGF secretion in TBI brains (Yang et al. 2022).

### Cortical spreading depolarization

TNS has been linked to the modulation of CSDs. In a preclinical SAH model, the application of TNS resulted in an inhibition of both CSDs and CSD-induced spreading ischemia (Shah et al. 2022). The trigeminovascular network is traditionally tied to the generation and pathogenesis of CSDs in migraine (Fregni et al. 2007). Clinically, however, chronic TNS in migraine results in the decrease of migraine symptomology, which has been ascribed to a potential effect on CSDs (Nash, Powell and White, et al. 2023; Magis et al. 2017; Didier et al. 2015; Russo et al. 2015).

### Cognitive function and wakefulness

TNS is linked to decreased cognitive dysfunction and increased wakefulness in TBI and loss of consciousness. To that end, the definition of wakefulness which we are using does not include measures of autonomic brainstem reflexes which are preserved in diseases of consciousness (Ma and Zheng, 2019; Hills 2010). TNS applied to a loss of consciousness model resulted in coma score improvement alongside reduced neurological dysfunction, indicative of improved recovery (Zheng et al. 2021). 7-day application of TNS to rats following severe TBI induction also decreases cognitive impairment, correlating with the decrease of amyloid precursor protein in the hippocampus (Xu et al. 2023). These pre-clinical results provide context for the clinical observation that TNS promotes arousal and functional recovery for patients with pDOC (Ma et al. 2023; Dong et al. 2022; Wu et al. 2022; Fan et al. 2019). 18-Fluorodeoxyglucose Positron Emission Tomography indicated significant hypermetabolism within the right parahippocampal cortex, right precuneus, and bilateral middle cingulate cortex in PVS patients undergoing TNS treatment (Ma et al. 2023). This indicated an increased arousal state and correlated with better functional recovery. Under healthy conditions, TNS enhances the signal-to-noise ratio of cortical neurons, allowing subjects to reach optimal discriminative performance in the oddball task at lower levels of neural activation (Tramonti Fantozzi et al. 2021).

### Neuropsychological function

TNS has been applied clinically to a wide array of psychological disorders, including major depression, PTSD, ADHD, and chemo brain. Daily usage of TNS, for at least 10 days, decreases symptomology in major depressive disorder and improves quality of life (Generoso et al 2019; Shiozawa et al 2014a, b; Cook et al 2013). In pediatric ADHD, it significantly decreases inattentiveness and hyperactivity (McGough et al. 2019). TNS also decreases the effects of chemo brain in women with breast cancer, resulting in less brain fog and emotional dysfunction (Zhang et al. 2020).

### Sensorimotor function

Clinically, TNS has been observed to decrease balance difficulties due to mTBI and MS (Tyler et al. 2019). Treatment in conjunction with physical rehabilitation results in a decrease of sensory dysfunction (Leonard et al. 2017). This was connected to an increase in BOLD activation in the bilateral premotor regions and the induction of neuroplastic changes. Modulation of the premotor regions is likely related to the beneficial effects of TNS following TBI + HS (Li et al. 2021a, b) and SAH (Shah et al. 2022) in rats, which also resulted in the preservation of motor function.

### Autonomic nervous system

#### Peripheral vasoconstriction

Mediated via actions upon the brainstem and ANS, TNS indirectly induces peripheral vasoconstriction (Powell et al. 2019). TNS activates the RVLM, C1, LC, and KF, inducing an upregulation of the sympathetic nervous system (SNS) (Peng et al. 2022; McCulloch et al. 1999). SNS upregulation induces peripheral vasoconstriction and increases BP. Subcutaneous TNS in naïve rats increases BP by ~ 9.2%, and by ~ 7.4% following TBI (Chiluwal et al. 2017). Successive stimulations in TBI rats result in further increasing the BP by 11.7%, preventing an eventual decrease in BP. In a model of severe HS, percutaneous TNS abrogated an ~ 70% decrease in BP, returning BP to ~ 67% of baseline (Li et al. 2019). This redirects perfusion towards the more metabolically demanding organs.

#### Cardiac performance

TNS indirectly modulates cardiac performance via the upregulation of the parasympathetic nervous system (PNS) (Li et al. 2019). In an HS model, TNS decreased SNS overactivity by 60% and increased PNS activity by 1.3x, as measured by heart rate variability. The modulation of both SNS and PNS directly affects HR and cardiac contractility, resulting in an overall maintenance of cardiac output (Marabotti et al. 2013). When the parameters of electrical TNS are specifically tuned, it can induce bradycardia, varying as a function of stimulus intensity (Shah et al. 2020). A similar response is seen with TNS induced by facial cooling, which resulted in an ~ 13% decrease of HR, alongside an increase in BP (Janczak et al. 2022). This was concurrent to an increase in left ventricle contractility and aortic compliance, indicating a balance between decrease of HR and increase in force of contraction (Prodel et al. 2023; Janczak et al. 2022;).

## Underlying mechanisms of trigeminal nerve stimulation

### Vasoactive neuropeptide regulation

#### Cerebral vasodilation

Activation of trigeminal sensory nerves results in the tri-fold release of vasoactive molecules, resulting in cerebral vasodilation (Fig. 3) (White and Powell et al. 2021; Goto et al. 2017; Mense 2010; Goadsby et al. 1988). The sensory nerves directly release neuropeptides in an antidromic manner, while communication via the facial nerve and sphenopalatine ganglion lead to parasympathetic release of vasoactive molecules from activated parasympathetic nerve fibers (White and Powell et al. 2021; Branston 1995; Goadsby and Edvinsson 1993; Lambert et al. 1984). Calcitonin gene-related peptide (CGRP), pituitary adenylate cyclase-activating peptide (PACAP), vasoactive intestinal peptide (VIP), nitric oxide (NO), substance P (SP), adenosine triphosphate (ATP), and neurokinin A are released during TNS (Messlinger 2018; Goto et al. 2017; Atalay et al. 2002; Gulbenkian et al. 2001; Uddman and Edvinsson 1989; Edvinsson et al. 1987). Of these agents, CGRP has been noted as the strongest vasodilatory neuropeptide, with efficacy at the femtomolar level, and is extant in the trigeminal nerve at high concentrations (Messlinger 2018; Goadsby 1993; Edvinsson et al. 1987). When CGRP is blocked via the local cortical application of h-CGRP, the observed increase in ipsilateral CBF (30 ± 6%) with TNS, is reduced by 50%, indicating that it is likely a major driving factor behind the cerebral vasodilatory effect (Edvinsson et al. 1998). NO, however, is the likely major effector released from the parasympathetic nerve fibers (Golanov et al. 2000; Golanov and Reis 1994). NO is released into the cerebrovasculature via intrinsic projections when the RVLM is activated and mediates endothelial-dependent vasodilation.

#### Cerebral anti-inflammation

While the exact mechanisms of the anti-inflammatory effects triggered by TNS are unclear, it is likely that the cerebral anti-inflammatory effects are related to the focal release of vasoactive neuropeptides within the brain (Fig. 3) (Shah et al. 2022; Li et al. 2021a, b(b)). CGRP is a negative inflammatory regulator, limiting tissue damage in inflammatory conditions (Borkum 2019; Holzmann 2013) and promoting anti-inflammatory phenotypes in animal models of MS (Rosseti et al. 2018) and cultured macrophages (Tang et al. 2017). PACAP has also been shown to have anti-inflammatory effects in the CNS (Waschek 2013), while VIP inhibits inflammation in a wide range of neurodegenerative disorders, including Alzheimer’s, Parkinson’s (PD), and Huntington’s diseases (Deng and Jin 2017). Finally, SP has been shown to mediate neuro-immune cell interactions, cytokine production, and immune cell proliferation rates (Mashaghi et al. 2016), and has anti-inflammatory properties in a mouse model of hind-limb ischemia (Kim et al. 2019).

### Neurotransmission

TNS modulates multiple neurotransmission pathways within the brain, including dopamine (Xu et al. 2023), gamma-aminobutyric acid (GABA) (Lauritsen and Silberstein 2019), glutamate, and hypocretin (Zheng et al. 2021) (Fig. 3). This modulation is likely the mechanism underlying the effects on CSD propagation, BBB function, psychological dysfunction, and consciousness with TNS.

#### N-methyl-d-aspartate receptors—glutamate and GABA

TNS modulates N-methyl-d-aspartate (NMDA) receptors in a bimodal fashion, in pathological or healthy conditions. In migraine, TNS downregulates NMDA receptors, resulting in a decrease of extracellular glutamate release (Lauritsen and Silberstein, 2019). This also results in increased GABAergic interneuron firing, thus modulating GABAergic signaling and lowering the firing rate of glutamate neurons (Buck et al. 2022). This potentially results in the prevention of a self-reinforcing cycle of glutamatergic kainite and NMDA receptor overactivation and further glutamate release, thus affecting CSD propagation (Nash, Powell, White, et al. 2023). EA-TNS in healthy conditions, however, increases NMDA receptor expression (Gong et al. 2022). This increases glutamate expression and transmission in the S1 cortex, resulting in decreased occludin expression and increased BBB permeability.

#### Dopamine and hypocretin

TNS has been observed to abrogate hippocampal dopamine dysfunction in preclinical TBI, resulting in a decrease of cognitive impairment (Xu et al. 2023). Activation of the trigeminal ganglion resulted in modulation of the dopamine transporter neurons in the substantia nigra pars compacta/ventral tegmental area, as well as modulation of the corticotropin-releasing hormone neurons of the paraventricular hypothalamic nucleus. Similar modulation of dopaminergic signaling may explain the effect of TNS in schizophrenia (Shiozawa et al. 2017); schizophrenia symptomology has been shown to be related to disruption in the interplay of glutamate and dopamine signaling in the cortex, midbrain, and striatum (Buck et al. 2022). The decreased firing rate of glutamate neurons projecting onto dopaminergic neurons would enable the homeostatic control of dopaminergic firing with TNS.

Modulation of dopaminergic signaling might also be interconnected to observed alteration of hypocretin signaling when TNS is applied to TBI (Zheng et al. 2021). Increased release of hypocretin, a neuroexcitatory peptide connected to wakefulness (Calipari and Espana, 2012), into the cerebrospinal fluid has been observed in a rat model of TBI with TNS application, alongside an increase in EEG activity (Zheng et al. 2021). The release of hypocretin regulates dopaminergic function and glutamatergic excitability, resulting in increased burst firing and tonic of dopamine neurons (Mehr et al. 2021; Calipari and Espana, 2012).

#### Creatine

Using MRI and MRS, unilateral transcutaneous TNS has been observed to decrease total creatine concentrations in the dorsolateral prefrontal cortex in healthy male humans (Ritland et al. 2023). In brain slices, creatine has been observed to inhibit neocortical pyramidal neurons (Bian et al. 2022) and is suggested to be a novel neurotransmitter. Its supplementation has been linked to improved cognitive processing in conditions characterized by both acute stressors (ie: exercise and sleep deprivation) and chronic, pathologic conditions (ie: aging, mTBI, Alzheimer’s disease, etc.) (Roschel et al. 2021). The decrease of creatine would, though, be in contradiction to TNS’ increase of cognitive function, indicating the necessity of further examination.

### Autonomic nervous system

#### Catecholaminergic signaling

Physiologically, SNS-induced catecholaminergic stimulation of α-adrenergic receptors in the smooth muscle results in vasoconstriction (Reid 1986). TNS modulates the SNS via activation of the RVLM, C1, LC, and KF (Panneton and Gan 2014; Burke et al. 2011; Panneton et al. 2006; Taylor et al. 2001; McCulloch et al. 1999). This induces the release of norepinephrine and neuropeptide Y (NPY) from sympathetic nerve terminals (Macarthur et al. 2011). Upon release, norepinephrine diffuses to smooth muscle target cells, whereupon it binds to α1 receptors and activates phospholipase C activity, thus promoting peripheral vasoconstriction. NPY further potentiates the contractile effects of norepinephrine by activating post-junctional Y1 receptors. TNS has been observed to increase the level of plasma norepinephrine following HS in rats, as a result of SNS modulation (Li et al. 2019). It also increases plasma NPY following TNS in healthy animals (Guo et al. 2021), indicating a direct linkage between the peripheral vasoconstriction induced by TNS and catecholaminergic modulation.

#### Cholinergic signaling

TNS’ modulation of cardiac function is mediated by cholinergic signaling (Moss et al. 2018; Gorini et al. 2010). TNS activates the NTS and DMN (Panneton and Gan 2014; Burke et al. 2011; Panneton et al. 2006; Taylor et al. 2001; McCulloch et al. 1999), which upregulates the PNS, as evidenced by heart rate variability measurement (Li et al. 2019). This results in the release of acetylcholine from parasympathetic postganglionic neurons (Gorini et al. 2010; Moss et al. 2018). During TNS, the acetylcholine binds to muscarinic receptors in the heart conduction system, thus producing a negative chronotropic effect, eliciting bradycardia (Gorini et al. 2010). In the spleen, acetylcholine stimulates α7-nicotinic receptors on macrophages, thus downregulating proinflammatory cytokine production (Pavlov 2019; Bonaz et al. 2017; Pavlov and Tracey 2015; Huston et al. 2006). This results in the production of a systemic anti-inflammatory phenotype, such as that observed in a pre-clinical HS model (Li et al. 2019). While it is possible that the aforementioned vasoactive neuropeptide regulation may have a bleed-through effect on systemic inflammation levels (Borkum 2019; Deng and Jin 2017; Holzmann 2013; Waschek 2013), the cholinergic anti-inflammatory pathway is more likely the cause behind a systemic anti-inflammatory response (Pavlov 2019; Pavlov and Tracey 2015).

## Future perspectives

### Key mechanistic gaps

TNS has been applied to a wide array of pathological conditions clinically and its underlying mechanisms have been increasingly studied in recent years (Fig. 1). Several mechanistic knowledge gaps, however, bear additional scrutiny, including bimodal and paradoxical effects in the brain, and unclear mechanisms for the systemic effects.

#### Bimodal actions on the blood brain barrier and neurotransmitters

The application of TNS in healthy and TBI conditions results in two distinctly opposing effects on the BBB (Gong et al. 2022; Chiluwal et al. 2017). In healthy conditions, TNS results in the increase of BBB permeability via the downregulation of occludin (Gong et al. 2022). In TBI, however, TNS decreases BBB permeability (Yang et al. 2022; Chiluwal et al. 2017), potentially via the increase of endothelial nitric oxide synthase expression, the production of endothelial NO, and the protection of cerebrovascular endothelium (Li et al. 2021a, b). Given the importance of BBB maintenance in both disease pathogenesis and therapeutic treatment of the cerebrovasculature, identifying the cause behind the bimodal modulation of BBB permeability is essential to ensure appropriate treatment.

TNS has been observed to downregulate glutamate activity in migraine via action on NMDA receptors (Lauritsen and Silberstein 2019). In healthy conditions, however, it has been observed to upregulate NMDA receptor expression (Gong et al. 2022). This results in an increase in glutamatergic signaling and the aforementioned decrease of occludin. The difference herein may lie in the application of TNS to healthy or pathogenic conditions, perhaps necessitating direct experimental comparison.

#### Unclear mechanisms and paradoxical observations in neuropsychological effects

While TNS is clinically applied to neuropsychological and cognitive dysfunctions, the actual underlying mechanisms are still fairly unclear (Zhang et al. 2020; McGough et al. 2019; Cook et al. 2016 and 2013; Trevizol et al. 2015; Schrader et al. 2011). While TNS’ clinical application in epilepsy has been supported with pre-clinical indications and mechanisms (Fanselow et al. 2000), its continued clinical growth and development in other conditions has not been mirrored with supporting pre-clinical research. The difficulty in modeling neuropsychological conditions in animals may have limited their accessibility. However, there are now extant models simulating conditions of chronic behavioral and neurological dysfunction in rodents (Angelucci et al. 2019; Sanchez et al. 2018). These could be used to assess whether the effects of TNS are solely down to the aforementioned effect on neurotransmitters, such as hypocretin and dopamine (Xu et al. 2023; Zheng et al. 2021), or whether there is another underlying mechanism in play.

There is also a possibly paradoxical observation that TNS results in the downregulation of creatine in healthy individuals (Ritland et al. 2023), while TNS has also been observed to increase cognitive function in chemo brain and pDOC (Ma et al. 2023; Zhang et al. 2020). Given that supplementation of creatine is linked to an increase in cognition, and its decrease is observed in diseases such as Alzheimer’s and PD (Roschel et al. 2021), a TNS-induced decrease in creatine would seemingly bely its positive effects. Additionally, given the increase in CGRP specifically in TNS, and its noted correlation to anxiety and depression in migraine and the chronic phase following cerebrovascular injury (Gungor and Pare 2014; Sink et al. 2011), it would likely benefit the field to assess whether its upregulation has negative long-term effects. While it is known for an intervention to have bimodal or paradoxical interactions in different conditions (Smith et al. 2012), identifying those conditions is critical in determining efficacious application of a treatment such as TNS. As such, it is necessary to broaden the preclinical application of TNS into the more chronic, long-lasting effects of acute/traumatic pathologies.

#### Systemic effects

The unique connections of the trigeminal nerve to the brain have led to an emphasis on the application of TNS in brain disorders. However, there is evidence that TNS also has systemic effects driven by ANS modulation (Li et al. 2019), most notably peripheral vasoconstriction, cardiovascular modulation, and systemic anti-inflammation. While it is apparent that the peripheral vasoconstrictive response is mediated via the SNS (Peng et al. 2022; McCulloch et al. 1999) and upregulation of norepinephrine (Li et al. 2019), the mechanisms underlying the modulation of cardiac performance and systemic anti-inflammatory effect are unclear. TNS presents a conditional effect on cardiac performance modulation, depending on stimulation parameters. Clinical TNS has little to no effect on HR and BP, being found safe to use chronically (McGough et al.^ 2019^; Ordas et al. 2020; Chou et al. 2019). Specially tuned electrical TNS (Shah et al. 2020), however, as well as TNS induced by facial cooling, both reduce HR and increase BP (Prodel et al. 2023; Janczak et al. 2022). This indicates that TNS has the capability to affect the cardiovascular system, via the upregulation of acetylcholine (Pavlov and Tracey 2015; Gorini et al. 2010). However, the exact cause of this effect is yet unclear; this makes it possible to activate cardiac modulation unintendedly when using new TNS parameters for therapeutic effect. This necessitates understanding which stimulation specifications generate these effects, and how to selectively exclude or induce them for maximal benefit.

Of TNS’ systemic effects, the anti-inflammatory one is the most unclear. The current understanding of the systemic anti-inflammatory effect of TNS is limited to ANS rebalancing (Li et al. 2019), however the deeper underlying mechanisms need further research. While it may be secondary to the controlled perfusion recovery, it may also be tied to the noted anti-inflammatory effects of direct ANS modulation (Abuelgasim et al. 2021). When the ANS is stimulated, as in TNS, impulses from the splanchnic and vagus nerves converge in the coeliac ganglion, activating the splenic nerve. This induces the release of norepinephrine and then acetylcholine, which inhibits the production of proinflammatory cytokines in macrophages possessing α7 nicotinic acetylcholine receptors (Ramos-Martinez et al. 2021). This anti-inflammatory reflex also results in release of pro-resolving mediators, the reduction of CD11b expression on neutrophils, maintenance of regulatory T cells, and the decrease of antibody secretion and B lymphocyte migration. TNS has already been shown to induce the release of both acetylcholine (Gorini et al. 2010) and norepinephrine (Li et al. 2019), indicating the likelihood of the above scenario in TNS and increasing the potential utility of TNS.

### Key methodological gaps

#### Tools limiting pre-clinical stimulation

The exact mechanisms underlying TNS’ effects in conditions such as migraine, epilepsy, and depression (Table 1) are still under debate. A refinement of the preclinical stimulation methodology could aid in investigation of TNS’ underlying mechanisms. For example, the mechanisms of TNS in even such a well-studied condition as migraine are limited to suppositions regarding ‘gate control’ and suprasegmental mechanisms (Coppola et al. 2022; Lauritsen and Silberstein 2019; Mehra et al. 2021). It is theorized, but not experimentally assessed, that the depolarization of Aβ fibers can inhibit transmission of action potentials via brainstem nociceptive fibers (Mehra et al. 2021). Given the well-known connection of the trigeminal nerve to migraine pathophysiology (Noseda and Burstein 2013), the lack of concrete understanding indicates the necessity of further chronic mechanistic research. Currently, the mechanistic understanding of migraine treatment with TNS is limited by the fact that it began in the clinical field. In fact, there are no extant pre-clinical studies which assess the effects of TNS in migraine (Tables 1–3). As such, the field is saturated with predominantly passive observations of TNS’ therapeutic effects, but not its mechanisms. Currently, however, there are no available electrodes which can be applied for chronic and/or awake stimulation in pre-clinical conditions. Unlike in humans, where the anatomy of the trigeminal nerve allows for transcutaneous stimulation (Vecchio et al. 2018; Chou et al. 2019; Magis et al. 2017; Didier et al. 2015; Reiderer et al. 2015), the anatomies of rodents (Dingle et al 2019) and swine preclude non-invasive stimulation (Salar et al. 1992). The development of implantable electrodes, such as the implantable cuff electrode used for VNS (Mughrabi et al. 2021), or a micro-technology-based 3D spiral electrode (Li et al. 2017) could overcome this limitation. Both electrode forms would potentially allow for chronic implantation and awake stimulation in pre-clinical conditions, thus permitting assessment of long-term efficacy in clinically reflective conditions. Not only would this allow for more effective assessment of the mechanisms in extant chronic pathological applications, but it would also allow for assessment of therapeutic effects in new applications such as chronic neurodegenerative and inflammatory diseases.

#### Translational safety considerations

TNS safety guidelines are well-established for chronic pathological conditions, however no guidelines are available for acute/traumatic settings. In chronic pathologies, safety concerns for TNS have been reserved to questions of effects on HR or BP, sedative effects, and localized paresthesia (Ptito et al. 2021; Lauritsen and Silberstein 2019; Chou et al. 2017). Similarly, in TBI, SAH, or polytrauma, ensuring the safety of TNS by minimizing complications is paramount. For example, in the hyper-acute phase of traumatic cerebral pathologies, where the initial injury is not yet managed, controlling the degree of cerebral vasodilation and CBF increase reduces the chance of bleeding and reactive damage (Saklani et al. 2022; Granger and Kvietys 2015; Broderick et al. 1994). While rodent models are appropriate for fast generation of data from a large cohort, they are not necessarily appropriate for cerebrovascular safety assessments (Eaton and Wishart 2017; Greek and Menache 2013). Currently, of the 27 pre-clinical manuscripts assessed herein, only one (Salar et al. 1992) used a large animal model (Tables 2 and 3). Although this study indicated that TNS retains its effect on CBF after SAH, the autologous blood injection model used was not suitable for studying clinical safety considerations, such as those in aneurysmal SAH (Salar et al. 1992). As such, there was no indication of a maximal CBF increase threshold to prevent reactive damage. New clinical safety guidelines for traumatic cerebrovascular conditions need to be established in existing large animal models (Eaton and Wishart 2017; Keifer and Summers 2016; Holm et al. 2016; Tasker et al. 2010).

#### Considerations for clinical translation

TNS is applied clinically for a number of chronic pathological conditions, however there is only one study currently available detailing the effects of clinical TNS usage in a traumatic condition. Results from the TRIVASOSTIM study indicate that external TNS at the ophthalmic branch neither decreased cerebral vasospasm or hydrocephaly nor did it improve functional outcome (Rigoard et al. 2023). This is in contrast to preclinical studies which found that TNS improved structural and functional outcome following SAH (Shah et al. 2022; Atalay et al. 2002; Salar et al. 1992). It is likely that this disjunction stems from issues regarding the stimulation strength and timing. Firstly, as mentioned by the investigators, TNS was kept under a certain threshold in order to diminish sensory feedback and maintain the double-blind (Rigoard et al. 2023). They clearly state that as the threshold is lower than that used clinically to treat migraine, it may have lessened the chance of beneficial effects. Additionally, it is implied that the stimulation was applied continuously, which would result in nerve desensitization and a loss in efficacy (Graczyk et al. 2018). The usage of a subtherapeutic stimulation threshold in conjunction with constant stimulation implies that TNS proceeded without an accurate understanding of the underlying mechanisms.

#### Refining the stimulation method

Optimizing the stimulation parameters and methodology would yield significant benefits for both clinical and pre-clinical research. The current knowledge regarding TNS indicates the existence of bimodal and paradoxical effects. While this phenomenon can be attributed to the observational conditions, such as healthy vs injured subjects, it may also be due to differences in stimulation parameters. It is evident that effects vary depending on the site of stimulation. Stimulation at the lingual branch lacks the increase in CBF observed with stimulation at V1 or V2 (Ishii et al. 2014; Sato et al. 1997). This might intimate that stimulation of the motor-sensory branch results in different effects than stimulation of the two purely sensory branches. Additionally, variation in BP response can be linked to stimulation site and method. Stimulation at the trigeminal ganglion has been associated with decreased BP (Goadsby et al. 1997), while stimulation at V1/V2 is generally associated with increased BP, if a change occurs (Prodel et al. 2023; Li et al. 2021a, b; Shah et al. 2020; Shiflett et al. 2015; Atalay et al. 2002). The only extant example of decreased BP occurring with V2 stimulation was very specific to an increase in stimulation amperage above 3.0 mA (Just et al. 2010). To that end, clinical TNS has no apparent effects on BP, though it is also delivered at V1 (McGough et al. 2019; Ordas et al. 2020; Chou et al. 2019). The operative difference therein may be the specific electrical parameters, as their variation can induce varying effects on BP, irrespective of stimulation site and method (Shah et al. 2020). Accordingly, the wide degree of variation in stimulation parameters might add to the observational disconnects. Looking at only the preclinical studies, all three branches and the ganglion have been targeted with stimulation frequency varying by as much as 300 Hz across only 27 manuscripts (Table 2 and 3). In order to limit observational disconnects and efficiently promote the usage of TNS across multiple clinical pathologies, the effects and specific stimulation parameters for each branch must be dialed in.

A more focused method of stimulation may be required to assess the effects of stimulation more accurately. While electrical, chemosensory, thermal, and mechanical stimulation have been used to safely target the peripheral trigeminal branches, they do so in an inexact manner, lacking the ability to target specific nerve fibers. Focused ultrasound possesses the ability to reversibly excite and suppress neural circuits at a submillimeter resolution, which may allow for more specified targeting and generation of isolated effects (Darrow 2019). The fibers comprising the trigeminal nerve are a mix of nociceptive (Aδ and C fibers) and low-threshold mechanoreceptors (Aα and Aβ fibers) (Gambeta et al. 2020), specific stimulation of which may generate different effects. While current clinical stimulation parameters are safe and effective for treatment of chronic pathological conditions (Table 1), acute/traumatic settings may present additional stressors which could increase the risk of adverse consequences. While the cerebral effects of TNS may be beneficial, an uncontrolled increase of CBF may lead to bleeding and reactive damage prior to initial injury management. As such, the ability to selectively abnegate these effects would be favorable. Given that cardiac regulation is linked to C-type nerve fibers (Pan et al. 1999), their specific isolation would be beneficial in this case.

A more focused stimulation method may also be beneficial in preventing activation of trigeminally-associated reflexes. Trigeminal activation is associated with the trigemino-cardiac reflex (TCR) (Schaller et al. 2009) and sudden unexpected death (SUD), including both sudden infant death syndrome (SIDS) and sudden unexpected death in epilepsy (SUDEP) (Vincenzi 2019; Singh et al. 2016; Matturi, 2005). TCR induction is linked to trigeminal mechanoreceptor activation (Meuwly et al. 2015; Schaller et al. 2009), and results in vagally-mediated asystole, bradycardia, and normo/hypotension, often to a detrimental outcome (Bassi et al. 2020; Meuwly et al. 2015; Panneton and Gan 2014; Panneton et al. 2006 and 2000; Taylor et al. 2001). SUD, on the other hand is linked to a low threshold for trigeminal activation combined with genetic/structural malformations regarding cardiorespiratory regulation (Lemaitre et al. 2015) which elicit uncontrolled and prolonged apnea and arrhythmia (Singh et al. 2016). Stimulating the trigeminal nerve without the risk of triggering the mechano-receptors, and thus TCR, or triggering the C-type fibers, and thus SUD in susceptible individuals, could increase its utility. This makes the usage of a more specific form of stimulation, such as focused ultrasound, of high importance.

### Diving reflex

The diving reflex (DR) is a unique, endogenous mechanism which overrides typical homeostatic reflexes and protects animals, including humans, from hypoxic conditions (Vega 2017; Davis et al. 2004; Schreer and Kovacs 1997; Elsner and Gooden, 1983; Goodwyn 1788). It is activated by a combination of TNS and apnea, and results in cerebral vasodilation, peripheral vasoconstriction, pulmonary vasodilation, splenic contraction, modulation of cardiac performance, obligate apnea, and systemic anti-inflammation and anti-oxidation. DR can be activated by breath-hold diving, facial cooling with apnea, facial immersion with apnea, nasopharyngeal stimulation, and tuned electrical TNS (Shah et al. 2020; Vega 2017; Davis et al. 2004). Of these induction methods, electrical TNS is easily usable in clinical settings, with a discreet and controllable dose–response relationship (Shah et al. 2020). The current forms of clinical TNS used for chronic brain disorders do not induce DR, as such stimulation parameters ought to be specifically tuned to induce DR (Shah et al. 2020). It could result in the modulation of hematologic and immune system components by splenic contraction (Eftedal et al. 2016; Palada et al. 2007; Bakovic et al. 2005) or the increase of pulmonary perfusion (Mélot and Naeije 2011; Davis et al. 2004). This extends TNS’ therapeutic applications into coagulation disorders or diseases of the pulmonary vasculature, while a systemic anti-oxidative effect could decrease the pathogenesis of chronic diseases which generate systemic oxidative stress, such as diabetes, cardiovascular diseases, and cancer (Sharifi-Rad et al. 2020).

### Untapped potentials of trigeminal nerve stimulation

#### Therapeutic intervention potentials

**Fig. 4:**
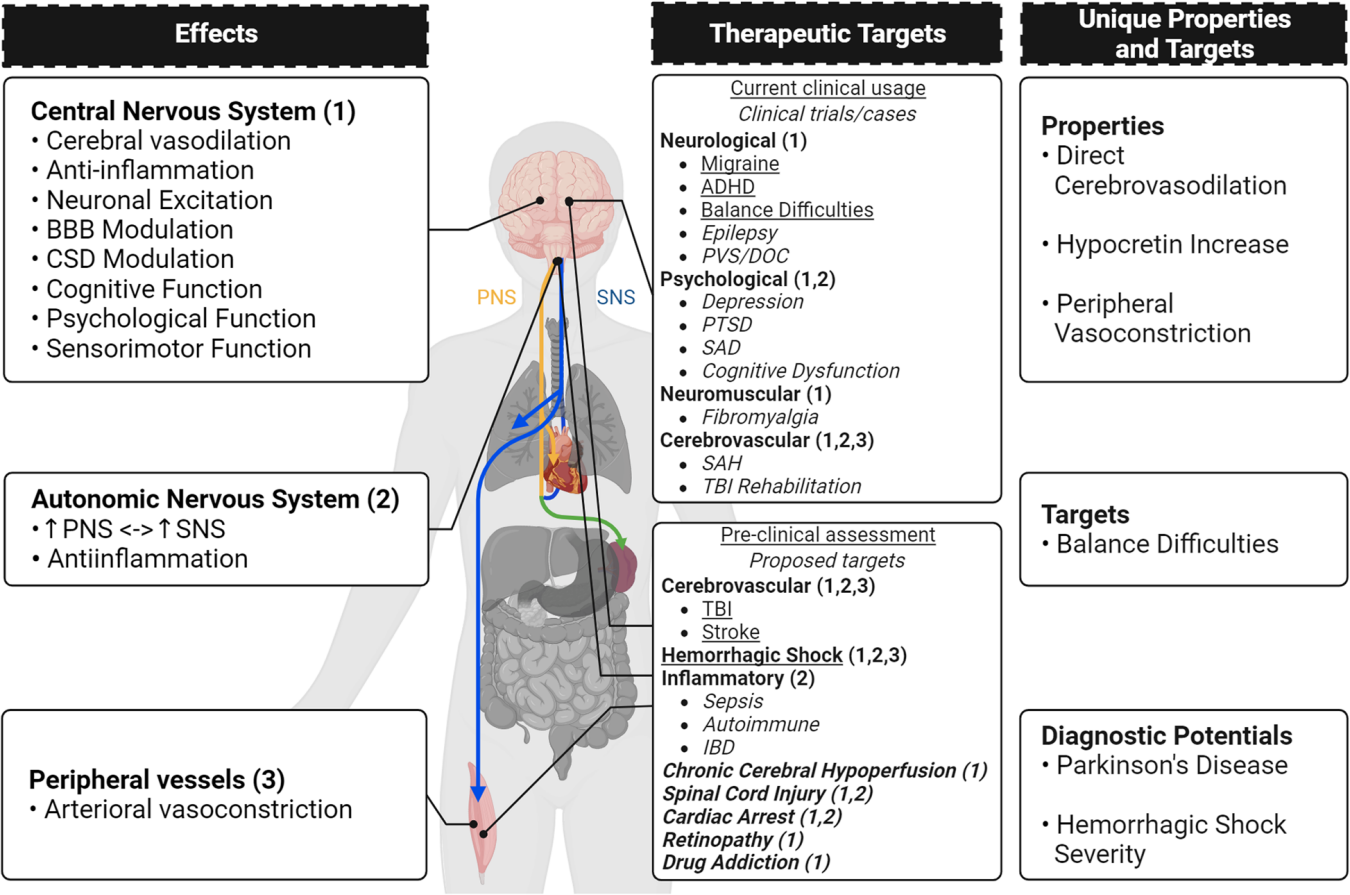
The effects, therapeutic targets, and unique properties of TNS. The effects of TNS can be separated into 3 main categories: effects on the CNS, the ANS, and the peripheral vasculature. Based on these effects, and particularly the effects on the CNS, many current therapeutic targets are focused on neurological, psychological, neuromuscular, and cerebrovascular pathologies. Already, some are approved for clinical usage, and others are in clinical testing. TNS is also currently under preclinical trials for acute/traumatic conditions, such as TBI, stroke, and hemorrhagic shock. Its multitudinous effects may allow it to be used therapeutically for the treatment of retinopathy, spinal cord injury, inflammatory dysfunctions, chronic cerebral hypoperfusion, and drug addiction. Setting it apart from other forms of bioelectronic medicine, TNS is able to cause direct cerebrovasodilation, increase hypocretin expression, and induce peripheral vasoconstriction. It is also the only one so far tried for balance difficulties due to mTBI. Uniquely, TNS also has diagnostic potentials in regard to Parkinson’s disease and hemorrhagic shock severity, which broadens its potential applications. (This figure was generated using BioRender.com) (ADHD: attention deficit and hyperactivity disorder; BBB: blood–brain barrier; CNS: central nervous system; CSD: cortical spreading depolarization; HS: hemorrhagic shock; IBD: irritable bowel disease; mTBI: mild traumatic brain injury; PNS: parasympathetic nervous system; PTSD: post-traumatic stress disorder; PVS: persistent vegetative state; SAD: social anxiety disorder; SAH: subarachnoid hemorrhage; SNS: sympathetic nervous system TBI: traumatic brain injury; TNS: trigeminal nerve stimulation)

The underlying mechanisms of TNS lend it the potential to be used therapeutically for a wide range of pathological conditions beyond its current scope (Fig. 4).

*Vasoactive Neuropeptide Regulation* The increase of vasoactive neuropeptides in the cerebrovasculature by TNS lends it broad therapeutic potentialities. This unique response of TNS induces significant cerebral microcirculatory dilation and can potentially be applied to global ischemia, such as in chronic cerebral hypoperfusion (CCH) (Fig. 4). In CCH particularly, loss of microcirculation leads to diffuse cellular degeneration (Du et al. 2017; Koshnam et al. 2017; Bandera et al. 2006). This is coupled with a slow, progressive increase in oxidative stress which leads to insidious hippocampal and white matter damage, and eventually vascular dementia. Currently, there is no effective treatment for the microcirculatory damage which causes CCH, thus making TNS’ increase of neuropeptides and induction of cerebral microcirculatory dilation a particularly attractive therapeutic option. TNS directly preserves microcirculation (Shah et al. 2022), thus avoiding potential side effects from systemic vasodilation. Additionally, the release of neuropeptides directly onto the cerebrovasculature bypasses the need for intact perfusion and is liable to work even in the case where the microvasculature is obstructed.

Reperfusion-injury following cardiac arrest (CA) may also be decreased by the modulation of vasoactive neuropeptides within the brain by TNS (Fig. 4). The rapid reperfusion and reoxygenation following CA-induced global cerebral ischemia generate a burst of reactive oxygen species and oxidative damage (Saklani et al. 2022; Granger and Kvietys 2015). In addition to vasoreactivity, the neuropeptides released by TNS have noted anti-oxidative effects (Borkum 2019; Rossetti et al. 2018; Tang et al. 2017; Deng and Jin 2017; Holzmann 2013; Waschek 2013). PACAP, CGRP, and VIP have all shown noted effects in cerebrovascular and neurologic diseases, such as SAH and PD. They are capable of decreasing reactive oxygen species production and increasing antioxidant levels, thus indicating a potential in abrogating post-resuscitative damage following CA.

Antidromic release of vasoactive neuropeptides by TNS may also be beneficial in retinopathy treatment (Fig. 4). Ischemic retinopathy, as seen in diabetes and hypertension, is the leading cause of preventable blindness (Ji et al. 2021). Damage to, or constriction of, the vessels that irrigate the retina may eventually lead to blindness. The lacrimal and ciliary sub-branches of the trigeminal nerve directly innervate the eye near the lacrimal gland and the ocular vasculature. As portions of the trigeminal sensory branches, these nerves experience antidromic release of vasoactive neuropeptides, potentially resulting in dilation of the nearby retinal vessels, thereby forestalling reactive damage. TNS may thus be able to prevent the progression of acute-stage retinopathy.

*Neurotransmission* TNS’ modulation of neurotransmitters, in particular dopamine, makes it a potential therapy for drug addiction (Fig. 4). It is known that dopaminergic signaling is fundamental in the different phases of addiction, including development, maintenance, withdrawal and relapse (Solinas et al. 2019). One of the current pharmacological treatment trends is the usage of pharmacological dopamine antagonists and agonists to disrupt the addiction cycle. TNS has been shown to downregulate dopaminergic firing (Xu et al. 2023), and may influence dopamine receptors (Zhou et al. 2003). SP, which is known to be released by TNS, has an attenuative effect on naloxone-precipitated withdrawal symptoms in rodents, when in the bioactive form of SP_(1–7)_ (Zhou et al. 2003). Treatment with SP_(1–7)_ reduces dopamine D2 receptor gene expression in the frontal cortex and nucleus accumbens (Zhou et al. 2003), a substrate of opioid withdrawal aversive effects (Harris and Ashton-Jones, 1994; Stinus et al. 1990; Koob et al. 1989). The increase of the SP bioactive heptapeptide SP (Hallberg and Nyberg 2003; Herrara-Marschitz et al. 1990) and the modulation of dopaminergic neurons and D2 receptors may allow TNS to be used as a dopamine-focused intervention in addiction treatment.

*Autonomic Nervous System* TNS’ modulation of the ANS may lend it therapeutic potential in spinal cord injury (SCI) and inflammatory disorders (Fig. 4). Traumatic SCI exhibits a penumbral zone which is exacerbated by perfusion decrease and loss of microvessels, which results in axon and oligodendrocyte loss (Khaing et al. 2018; Muradov et al. 2013). The activation of the RVLM, C1, and LC by TNS upregulates the SNS (Panneton and Gan 2014; Burke et al. 2011; Panneton et al. 2006; Taylor et al. 2001; McCulloch et al. 1999), which increases norepinephrine, resulting in a concurrent increase in central perfusion (Li et al. 2019). Given that the cervical and lumbar arteries are fed by the basilar artery and aorta, respectively (Biglioli et al. 2004), increasing central perfusion can improve spinal perfusion. The re-establishment of proper SNS and PNS function by TNS may also be beneficial in inflammatory disorders such as irritable bowel disease, sepsis, and autoimmune diseases (Pavlov 2019; Bonaz et al. 2017; Pavlov and Tracey 2015; Huston et al. 2006). Altered balance between the SNS and PNS negatively affects the immune system, however TNS has shown the capability to rebalance the ANS (Li et al. 2019). The resultant modulation of the inflammatory reflex arc by TNS may lend it a systemic anti-inflammatory effect.

#### Diagnostic potential

TNS has potential as a diagnostic tool for both PD and HS severity (Fig. 4). Stimulation of the trigeminally innervated nasal region with either eucalyptol (Tremblay et al. 2017) or v/v CO_2_ (Barz et al. 1997) has been shown to be efficacious in diagnosing olfactory dysfunction (OD) due to PD. While OD is typically associated with reduction in trigeminal sensitivity, studies suggest that patients with OD due to PD have levels of trigeminal sensitivity more associated with healthy individuals (Tremblay et al. 2017; Barz et al. 1997). In fact, TNS-induced olfactory event related potentials did not differ between healthy controls and PD patients (Barz et al. 1997). Altogether, these results imply that PD patients present with a specific pattern of chemosensory impairment, typified by olfactory impairment and trigeminal intactness (Tremblay et al. 2017; Barz et al. 1997). This suggests that the application of TNS in olfactory testing may be beneficial as a screening tool for PD.

During application of TNS to the treatment of HS in rodents in our lab (Li et al. 2019), we observed that the severity of HS correlated with the intensity of TNS required to increase BP (data not published). This is likely related to the activation of the RVLM that occurs in HS (Ranjan and Gulati 2023). During hemorrhage, the RVLM and NTS activate, inducing upregulation of the SNS, peripheral resistance, and heart rate, resulting in preservation of central blood flow; this is termed the endogenous compensatory pressor response. Adrenergic C1 neurons in the RVLM are the primary drivers of the response; they regulate SNS activity controlling heart rate and peripheral resistance (Souza et al. 2022). As hemorrhage continues, the endogenous response shifts from compensation to decompensation, characterized by a decrease of C1 neuron and SNS activity, in a last-ditch effort to reduce cardiac metabolism and increase venous return. Additional stimulation of the RVLM C1 neurons during the compensatory response to hemorrhage does not significantly increase lumbar sympathetic nerve activity, and thus does not prevent decompensation. As TNS’ effect on BP functions via the RVLM (Peng et al. 2022; McCulloch et al. 1999), it is reasonable to infer that this same ceiling effect comes into play during TNS treatment of HS. The stronger the extant compensatory response, the lower response to additional RVLM C1 neuron activation is, resulting in a seemingly weaker effect of TNS on BP. This relationship between this ceiling effect and TNS’ effect on peripheral resistance and BP may act as a method to clinically gauge how severe a case of HS is upon presentation.

## Conclusion

Over the course of 49 years, knowledge of TNS has grown widely. The unique connections of the trigeminal nerve allow TNS to activate powerful mechanisms, including the modulation of neuropeptides, neurotransmission, and the ANS. This engenders a powerful suite of effects which has supported the clinical application of TNS to the treatment of many chronic conditions. The induction of cerebral vasodilation in the micro- and macro-vasculatures increases oxygen to injured tissue in the hyper-acute phase of traumatic conditions, preserving at-risk tissue. Modulation of hypocretin production regulates dopaminergic and glutamatergic signaling, upstream of factors on CSDs and the BBB, in addition to the well-known effects on consciousness. The two in conjunction decrease the development of brain damage, the benefits of which are compounded by the decrease of neuroinflammation, without the necessity of systemic administration. As such, TNS may be particularly impactful in the hyper-acute phase of primary brain injury, such as ischemic stroke, as well as conditions exhibiting secondary brain injury, such as cardiac arrest. The trigeminal nerve’s connections to the olfactory system and RVLM lend TNS diagnostic potential in conditions such as Parkinson’s disease and acute shock. In order to achieve these therapeutic and diagnostic possibilities, however, the underlying mechanisms must be fully clarified, and the methodological gaps be filled. TNS’ sheer breadth of potential as a therapeutic modality and diagnostic tool renders it a promising field of translational medicine.

## Data Availability

Not Applicable.

## Abbreviations

ADHD: Attention deficit and hyperactivity disorder
ANS: Autonomic nervous system
BBB: Blood–brain barrier
BP: Blood pressure
C1: Catecholaminergic regions
CBF: Cerebral blood flow
CGRP: Calcitonin gene-related peptide
CNS: Central nervous system
CSD: Cortical spreading depolarization
CVR: Cerebrovascular resistance
DMN: Dorsal motor nucleus
DR: Diving reflex
EA-TNS: Electroacupuncture TNS
GABA: Gamma-aminobutyric acid
HS: Hemorrhagic shock
KF: Kölliker-Fuse
LC: Locus coeruleus
MAP: Mean arterial pressure
MDH: Medullary dorsal horn
mTBI: Mild TBI
NMDA: N-methyl-d-aspartate
NO: Nitric oxide
NOS: Nitric oxide synthase
NPY: Neuropeptide Y
NTS: Nucleus tractus solitaries
OD: Olfactory dysfunction
PACAP: Pituitary adenylate cyclase-activating peptide
PD: Parkinson’s Disease
pDOC: Prolonged disorders of consciousness
PNS: Parasympathetic nervous system
PTSD: Post-traumatic stress disorder
RN: Raphe nuclei
RVLM: Rostral ventrolateral medulla
SAH: Subarachnoid hemorrhage
SIDS: Sudden infant death syndrome
SNS: Sympathetic nervous system
SP: Substance P
SUD: Sudden unexpected death
SUDEP: Sudden unexpected death in epilepsy
TBI: Traumatic brain injury
TCR: Trigemino-cardiac reflex
TMN: Trigeminal mesencephalic nucleus
TNS: Trigeminal nerve stimulation
V1: Ophthalmic branch of the trigeminal nerve
V2: Maxillary branch of the trigeminal nerve
V3: Mandibular branch of the trigeminal nerve
VIP: Vasoactive intestinal peptide
VNS: Vagus nerve stimulation
